# Not all fun and games: Disparities in school recess persist, and must be addressed

**DOI:** 10.1016/j.pmedr.2023.102301

**Published:** 2023-06-25

**Authors:** Hannah R. Thompson, Rebecca A. London

**Affiliations:** a University of California, Berkeley, School of Public Health, 2121 Berkeley Way, 6120, Berkeley, CA 94720-7360, USA; b University of California, Santa Cruz, Sociology Department, 202 Rachel Carson College, Santa Cruz, CA 95064, USA

**Keywords:** Recess, Schools, School health, Physical activity, Mental health, Academic performance

## Abstract

School recess is an evidence-backed approach to increase school-based opportunities for students to play, accrue necessary physical activity, and socialize with peers, to the benefit of their physical, academic, and socioemotional health. As such, the Centers for Disease Control recommend at least 20 min of daily recess in elementary schools. However, unequal provision of recess contributes to persistent health and academic disparities for students, which remain to be addressed. We analyzed data from the 2021–22 school year from a sample of low-income (Supplemental Nutrition Assistance Program Education-eligible) elementary schools (n = 153) across California. Just 56 % of schools reported providing more than 20 min of recess daily. Differences in daily recess provision were apparent, with students in larger and lower-income schools receiving less daily recess than students in smaller and higher income schools. These findings support the enactment of legislation mandating health-sufficient daily recess in California elementary schools. They also highlight the importance of, and need for, annually-collected data sources to enable monitoring of recess provision, and potential disparities, over time, in order to assist in identifying additional interventions to address this public health problem.

## 1 Introduction

Both the American Academy of Pediatrics and the Centers for Disease Control and Prevention have issued statements on the importance of school recess (Murray and Ramstetter, 2013, CDC and SHAPE America, 2017) and recommend that all children have 20 min or more of daily recess. Underlying these recommendations is a deep research base from health, public health, social science and education documenting the multiple benefits that children accrue from this unstructured playtime at school. For instance, playing at recess helps children to concentrate in class (Jarrett et al., 1998, Pellegrini and Davis, 1993), augments their executive functioning (Massey et al., 2021), and improves classroom behavior (Barros et al., 2009, Massey et al., 2021) as well as academic performance (Rasberry et al., 2011, Waite-Stupiansky and Findlay, 2002). It is also an important place where students learn and practice social-emotional skills, such as conflict resolution, teamwork, empathy, and self-regulation (Miyamoto et al., 2015, Massey et al., 2017). Studies have shown that a high-quality recess environment is associated with improvements in students’ school attendance (Leos-Urbel and Sanchez, 2015) and overall school climate (London et al., 2015). And, as the only opportunity for free play in the school day, it is an important contributor to children’s abilities to accrue the 60 min of physical activity during the school day recommended by the Institute of Medicine (Institute of Medicine, 2013).

Yet, evidence suggests that students have had persistent unequal access to this important developmental time. First, students living in certain communities have had historically less recess. Barros, Silver and Stein (2009) analyzed data from the 1998–99 cohort of the Early Longitudinal Childhood Survey and found that while 77 % of White children had some recess, just 41 % of Black children and 62 % of Hispanic children had some recess. Disparities were similarly found for children of parents with less education and lower income and those living in larger and middle-sized cities. A 2009 Gallup survey of school principals (RWJF, 2010) that was re-analyzed by London (2019) similarly documents disparities in recess provision, finding that even when recess is scheduled regularly, students in lower-income and urban schools have less access (in terms of days/week or minutes/day) than those in higher-income suburban and rural schools. Geographic variations in recess provision have also been demonstrated, with East South Central and West South Central US schools less likely to offer 20 min or more of recess daily compared to the national average (Turner et al., 2013). More recently, Monnat et al (2017) found that Nevada public schools with a higher proportion of Black students had a greater odds of withholding recess for disciplinary reasons and lower odds of having recess supervisors who are trained to promote physical activity.

While a national, state-level recess policy classification system exists (National Cancer Institute, 2023, https://class.cancer.gov/) and is publicly available, there is no annually-collected data source that comprehensively compiles national recess provision data, precluding our ability to monitor recess minute provision and policy adherence, and potential disparities, over time. In the absence of national tracking systems, using existing state and/or local data can help us better understand the current state of recess, which can help address potential disparities in provision more locally. To determine if disparities in recess provision persisted in the largest and second-most diverse state in the nation, we analyzed recess data collected by California local health departments from low-income elementary schools during the 2021–22 school year. These data can help us identify if, and what type, of work is necessary to ensure students have access to this important public health intervention.

## 2 Methods and results

We analyzed data that were collected by California local health departments for the 2021–22 school year from a sample of low-income (Supplemental Nutrition Assistance Program Education-eligible) elementary schools (n = 153) across the state that answered a question related to recess time (91 % of schools surveyed). Schools were asked, “In addition to a lunch break, recess is provided for all students” with answer options never, <10 mins/day, 10–19 mins/day, 20 mins/day, and >20 mins/day. Data were primarily principal-reported. Publicly available school-level demographic data were downloaded from the California Department of Education. The school-level proportion of students by race/ethnicity was dichotomized as White (% non-Hispanic White students) or non-White (% of students identifying as any other race/ethnicity other than non-Hispanic White). Because this study did not include human subjects and was not considered systematic research designed to contribute to generalizable knowledge, it did not require a human subjects research review by the university Institutional Review Board.

The majority of sample schools (82 %) were grades K-5 or K-6, with the remaining schools (18 %) grades K-8. Average enrollment was 441 ± 198 students per school, with, on average, 62 % Hispanic, 8 % non-Hispanic African American, 4 % non-Hispanic Asian, and 18 % non-Hispanic White student enrollment. Sample schools had significantly more non-White students (82 % vs. 78 %; p < 0.001) and students who qualify for free or reduced-price meals (FRPM), a proxy for socioeconomic status (77 % vs. 59 %; p < 0.001) than non-sample elementary schools (n = 5,501) in the state.

Based on descriptive statistics, just 56 % of schools reported providing more than 20 min of recess daily; 16 % provided 20 mins/day and 27 % provided 10–19 mins/day. A logistic regression model accounting for clustering by school district and adjusted for overall, FRPM, and non-White student enrollment, shows that larger schools and schools with greater than 50 % FRPM enrollment have lower odds of providing more than 20 min of recess daily (Table 1).

**Table 1:** Odds ratios^A^ for providing more than 20 min of recess/day among a sample of California elementary schools (n = 153) during the 2021–22 school year.

	Odds Ratio ± Robust SE	95 % Confidence Interval
Schools with ≥50 % FRPM enrollment	**0.10 ± 0.11**	**0.012, 0.825**
Enrollment per 100 students	**0.79 ± 0.10**	**0.620,0.999**
Proportion of non-White student enrollment	0.39 ± 0.37	0.062, 2.480

A Estimates derived from logistic regression model accounting for clustering by school district and adjusted for overall, Free and reduced-price meal (FRPM), and non-White student enrollment.

For every 100-student increase in enrollment, the odds of providing more than 20 min of daily recess declines 21 %. Schools with ≥50 % FRPM student enrollment have a 90 % lower odds of providing more than 20 min of daily recess compared to schools with <50 % FRPM enrollment, though this finding should be interpreted cautiously, as only 9 % of sample schools had <50 % FRPM enrollment. Still, these findings demonstrate disparities in daily recess provision, with students in larger and lower-income schools receiving less daily recess than students in smaller and higher income schools.

## 3 Discussion

These findings continue to support the need for state reforms to support daily recess for all children. Schools in states with recess laws are more likely to provide recess (Slater et al., 2012) and students in states with recess laws are more likely to be physically active every day (Clevenger et al., 2022). In recent years, states such as Arizona, Florida, Missouri and New Jersey have enacted legislation that requires a certain number of minutes for scheduled recess (National Association of State Boards of Education, 2023). California has introduced such legislation in Senate Bill 291, which requires 30 min of daily recess for students in grades K-6 (California State Legislature, 2023). Passage of this legislation is an important first step in ensuring students across California, regardless of background, have daily recess. This bill is particularly timely as it provides an important opportunity for schools to scaffold non-academic supports to help students recover losses in both socio-emotional health and physical activity brought on by COVID-19 pandemic school closures (Hoffman and Miller, 2020).

Still, even in states with laws that mandate recess minutes, there is no system of accountability for adherence, or even one that collects data to track how schools implement these policies. In Arizona, after a state recess bill was passed in 2018, a study found law adherence was low across the state (Griffo et al., 2022). While passage of the law signaled the recognized importance of recess, without provisions in the law to support schools in addressing the known barriers to recess provision, and without a system for tracking accountability to the law, many schools simply didn’t comply (Griffo et al., 2022). In California, the lack of an accountability system for physical education legislation resulted in 127 school districts, collectively educating over half the students in the state, being sued for non-compliance (Thompson et al., 2018). To be most effective, policies that mandate recess must include systems of support and accountability to ensure schools are able to successfully comply with the law.

Limitations to this brief analysis warrant mention. These data represent a sample of low-income K-5/6 and K-8 schools in California and findings may not necessarily be generalizable to higher income schools or schools in other states. In addition, data on other school-based physical activity or socio-emotional health improvement opportunities (like physical or health education) were not available/included. Finally, while California’s Senate Bill 291 would require 30 min of daily recess, the recess question used in this study had “>20 min of recess/day” as the top category, precluding our ability to specifically assess the proportion of schools currently receiving 30 min of recess/day.

School recess is an evidence-backed approach to increase school-based opportunities for students to play, accrue necessary physical activity, and socialize with peers, to the benefit of their physical, academic, and socioemotional health. Despite the known benefits of recess, disparities in provision continue to persist. The data presented in this article offer a snapshot of what we can presume are even larger differences between higher and lower income, and larger and smaller schools across California. Enacting legislation to support recess provision is critical, as is providing the necessary supports for schools to implement recess successfully and in accordance with the law. Finally, improvements in recess minute surveillance and monitoring nationwide would improve our ability to track these disparities across locales and over time, as well as strengthen our ability to identify programmatic and policy interventions to best address this public health problem.

## CRediT authorship contribution statement

**Hannah R. Thompson:** Conceptualization, Data curation, Formal analysis, Methodology, Writing – original draft, Writing – review & editing. **Rebecca A. London:** Conceptualization, Writing – original draft, Writing – review & editing.

## Declaration of Competing Interest

The authors declare that they have no known competing financial interests or personal relationships that could have appeared to influence the work reported in this paper.

## Data Availability

Data will be made available on request.
